# Effect of three-body wear on the surface properties of different polymer-based CAD/CAM dental restorative and prosthetic materials

**DOI:** 10.1186/s12903-026-09028-5

**Published:** 2026-07-04

**Authors:** Abdallah Fawzy Elsadany, Hesham Samy Borg, Enas A. Elshenawy

**Affiliations:** 1 Faculty of Dentistry, Alsalam University, Tanta, Egypt; 2 Faculty of Dentistry, Tanta University, Tanta, Egypt

**Keywords:** Three-body wear, Microhardness, Chewing simulator, Surface roughness, CAD/CAM

## Abstract

**Background:**

Computer aided designed/ computer aided manufactured (CAD/CAM) materials offer diverse options for prosthetic rehabilitations, yet correlation between materials’ surface properties and aging through chewing simulation needs characterization. The aim of this study is to measure and evaluate the effect of three body wear on three CAD/CAM materials (poly methylmethacrylate (PMMA), composite resin and polyether ether ketone (PEEK)) regarding their microhardness and surface roughness.

**Methods:**

Seventy-two disc-shaped specimens (20 × 6 mm) were fabricated (*n* = 24/ group) from PMMA (Yamahachi PMMA Disk), Nano-ceramic Composite (Grandio Blocs, VOCO), and PEEK (BioHPP, Bredent). Specimens were subjected to 120,000 chewing cycles in a chewing simulator using a 3-body wear protocol with a pumice slurry. Wear volume (mm^3^) was measured digitally. Surface roughness (µm) and Vickers hardness (VHN) were evaluated twice: at baseline and within the wear facets post-simulation. Data were analyzed using Two-way and One-way ANOVA (*P*-value < 0.05), and Pearson correlation was used to measure the relationship between properties.

**Results:**

One-way ANOVA revealed a significant difference between material types (*P* = 0.000*), with composite exhibiting the lowest mean volume loss (0.08 mm^3^), significantly outperforming PEEK (0.23mm^3^) and PMMA (0.55 mm^3^). For surface roughness, a significant interaction was observed between material type and wear simulation (*P* = 0.000*), while there was no significant interaction between them on surface hardness (*P* = 0.89). Composite significantly outperformed PEEK and PMMA regarding all tested properties.

**Conclusion:**

The 3-body wear simulation induced significantly different volumetric loss across all groups, with the magnitude of loss largely dependent on the material’s specific composition and baseline hardness. In addition, the surface roughness and microhardness of three investigated materials were considerably impacted by three-body wear. PMMA have the lowest wear resistance, lowest microhardness, and the greatest surface roughness both before and after wear, while composites with high filler loading and high microhardness have the highest wear resistance followed by PEEK. After wear, the surface roughness of the composite is acceptable.

## Background

One important factor that affects the longevity, appearance, and hygiene of restorative and prosthetic dental materials is wear [1]. Reduced wear resistance promotes plaque accumulation, which leads to restoration discoloration and aesthetic issues. Also, increased wear decreases the vertical dimension of dental restorations and reduces masticatory efficiency; it may also cause fatigue of the masticatory muscles, which can lead to temporomandibular joint disorders [2–5].

Abrasive wear is commonly distinguished into two principal modes: two-body and three-body abrasion [6, 7]. Two-body wear refers to the physiological loss of dental hard tissues resulting from direct tooth-to-tooth contact without the presence of intermediate particles, leading to localized wear at occlusal contact areas. In contrast, three-body wear occurs when an intervening slurry of abrasive particles is present between two contacting surfaces. In this condition, the applied pressure is transferred to the abrasive particles, which subsequently abrade the surface asperities and contribute to material removal [8]. 

In contrast to two-body wear, three-body wear provides a more faithful representation of intraoral conditions, since mastication typically occurs with food particles, salivary constituents, or other debris interposed between opposing surfaces [9, 10]. The presence of an intermediate medium dissipates occlusal forces and reduces the severity of direct abrasion, resulting in a wear pattern that more closely mirrors clinical performance [11, 12].

In dental materials, microhardness testing provides valuable insight into the surface integrity, structural homogeneity, and potential clinical durability of restorations [13]. However, excessively high hardness may result in increased wear of the opposing dentition or restorative counterparts. Therefore, achieving an optimal balance between microhardness and wear resistance is essential to ensure long-term clinical performance, surface smoothness, and preservation of occlusal harmony in restorative/ prosthetic dentistry [14–16].

The mechanical behavior, aesthetic quality, and long-term clinical performance of dental restorative and prosthetic materials are all greatly impacted by surface roughness, a crucial surface characteristic. It refers to the microscopic irregularities that exist on the material’s surface, which are commonly quantified using parameters such as the average roughness (Ra) value [17]. It plays a pivotal role in determining plaque accumulation, discoloration, gloss retention, and resistance to wear. A smoother surface promotes favorable optical characteristics and reduces bacterial adherence, while an irregular surface accelerates biofilm formation, mechanical degradation, and surface fatigue under masticatory stress [18, 19].

The surface finish of restorative materials plays a crucial mechanical role in determining their functional longevity in addition to being an aesthetic factor [20, 21].

The evolution of computer-aided design and computer-aided manufacturing (CAD/CAM) systems has significantly transformed the scope of restorative and prosthetic dentistry. Polymer-based materials such as polymethyl methacrylate (PMMA), nano-ceramic CAD/CAM composite materials, and polyetheretherketone (PEEK) have emerged as integral components of digital fabrication workflows. Through industrial polymerization and precision milling, these materials provide mechanical, biological, and esthetic advantages that extend beyond the limitations of conventionally processed resins including polymerization shrinkage, internal porosities, lower mechanical properties, and increased wear susceptibility [22–24]. PMMA-based CAD/CAM materials are widely used for provisional restorations, occlusal splints, and denture bases, while CAD/CAM composite resins are commonly used for definitive restorations such as crowns, inlays, onlays, and veneers [25]. In addition, PEEK has been introduced for prosthetic frameworks, implant-supported restorations, and removable partial denture frameworks because of its high strength, chemical stability, and favorable biocompatibility. PMMA offers economical fabrication, good esthetics, and ease of replacement; CAD/CAM composites deliver optimal flexibility, repairability, and lifelike optical properties; and PEEK provides superior mechanical endurance and implant compatibility. Together, these materials underscore the evolution of digital prosthodontics toward minimally invasive, reproducible, and patient-specific restorative approaches [26–28].

Although CAD/CAM fabrication may improve material homogeneity and mechanical performance, limited information is available regarding the wear behavior of these materials under three-body abrasive conditions simulating mastication. Despite their diverse primary clinical applications—ranging from temporary provisionals to definitive restorations—these materials increasingly compete within the digital workflow as full-contour occlusal surfaces for long-term interim prostheses. The selection of these materials represents three distinct CAD/CAM polymer classes: linear unfilled thermoplastics (PMMA), high-performance reinforced polymers (PEEK), and resin-nanoceramics (Composite). Comparing these distinct structural strategies under identical standardized wear protocols allows for a fundamental understanding of how different filler and matrix configurations withstand abrasive functional cycles. Consequently, this provides clinicians with objective data on occlusal stability and surface durability during extended treatment phases where maintaining the vertical dimension of occlusion is clinically critical.

Therefore, the present study aimed to evaluate the wear behavior and the resulting changes in surface roughness and microhardness specifically within the resultant wear facets of different CAD/CAM materials (PMMA, composite resin and PEEK) following three-body wear simulation.

The tested null hypotheses will be: (1) There will be no significant difference between the groups regarding the wear volume. (2) There will be no significant difference in surface roughness of each CAD/CAM PMMA, composite and PEEK materials before and after three-body wear. (3) There will be no significant difference in microhardness of all groups before and after three-body wear.

## Materials and methods

Three CAD/CAM materials used in this study were listed in Table 1.

**Table 1:** Detailed description of the materials used in the study

Material type	Commercial Name	Chemical composition	Manufacturer
CAD/CAM PMMA	Yamahachi PMMA Disk	PMMA + TiO₂ + Fe₂O₃ + Carbon black (pigments). No filler	Yamahachi Dental Mfg. Co., Ltd., Gamagori City, Japan
CAD/CAM composite	Grandio Blocs (GR)	86% nanohybrid filler and a 14% resin matrix made of UDMA and DMA monomers.	Voco, Cuxhaven, Germany
CAD/CAM PEEK	BioHPP (Bredent)	20% nanoceramic filler & ZrO_2_, 80% PEEK	bredent GmbH, Weissenhorner, Germany

### Study design

**Fig. 1:**
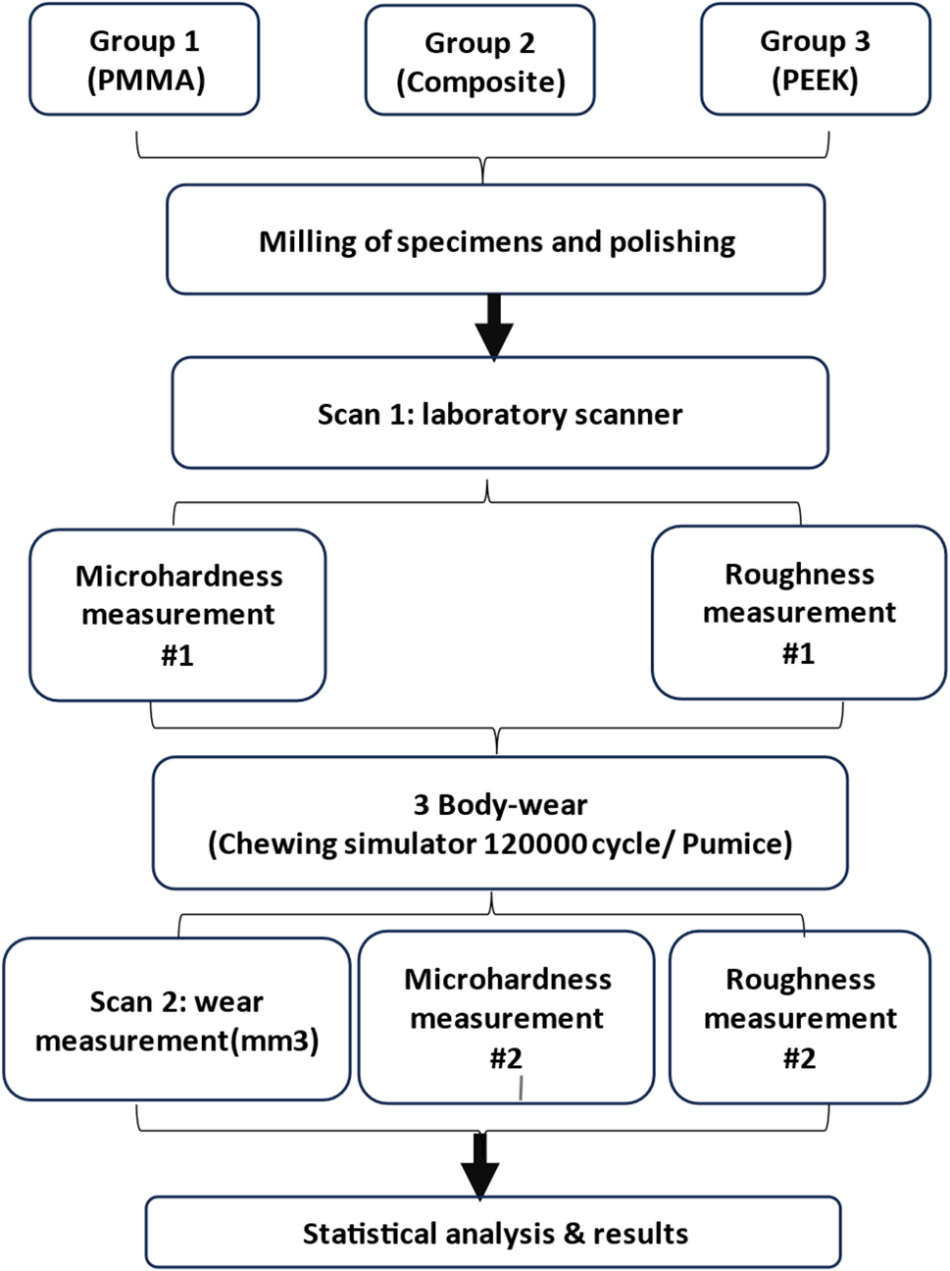
Flow chart demonstrating the study design

Three CAD/CAM materials; PMMA, Composite and PEEK were compared by evaluating their surface properties. Each specimen served as its own control, surface roughness and microhardness were first measured at baseline. The specimens then underwent a 3-body wear challenge (120,000 cycles). Following this, measurements were repeated strictly within the boundaries of the resulting wear facets to quantify the specific impact of the chewing simulation on the material’s surface properties Fig. 1.

The study was approved by the Research Ethics Committee, Faculty of Dentistry, Alsalam University, under approval number [Sue 013010253].

GPower, version 3.1.9.2, was used to determine the sample size. Based on the findings of an earlier study, the calculated impact size is 0.463 [29]. A significance level of 5% and a power of 95% were used to estimate the sample size. Each group has an estimated sample size of 24 samples.

### Specimens preparation

The materials were supplied as CAD/CAM blocks. Specimens (20 mm diameter, 6 mm thickness) of each material group were designed using Fusion ICAM5 software (ICAM Technologies Corp, Canada) to ensure a secure fit within the customized holders of the dual-axis chewing simulator and to provide a sufficiently large, flat surface area for accurate volumetric scanning and profilometric measurements and to ensure structural rigidity, preventing any flexure or bulk fracture during the abrasive loading [30] The specimen geometry was optimized to ensure reproducible positioning and accurate digital subtraction, following the general validation principles for wear simulation described by Heintze et al., [31]. Digital designs were exported in STL format then milling was performed using a 5-axis high-precision milling machine (CORiTEC 350i, Imes-Icore GmbH, Eiterfeld, Germany) under constant water cooling to prevent thermal degradation of the polymer matrices. The specimens were polished using Sof-Lex discs (3 MESPE, Seefeld, Germany) in a decreasing order of abrasiveness till the ultrafine (8 μm) using a low-speed hand piece (4.000–5.000 rpm) after being finished with 600 and 1200 grits silicon carbide paper [29]. Before the test, the polished surfaces were rinsed with water, cleaned in an ultrasonicator to get rid of any remaining surface debris. Subsequently, specimens were immersed in distilled water for seven days at 37 °C to achieve water sorption equilibrium, followed by storage at room temperature for 24 h to standardize the environmental state. This pre-conditioning ensures that the polymer-based materials reach a stable dimensional and mass state, preventing initial water uptake from confounding the volumetric wear measurements [32]. The polishing protocol was followed for all specimens to minimize variability in baseline surface roughness. However, slight differences in initial roughness may occur due to inherent variations in material composition and polishing behavior; therefore, comparisons were primarily performed within each material group before and after the wear simulation.

Orientation grooves were manually placed on the sides of specimens for digital matching of each sample and calculation of wear volume. Standardization was achieved by defining three equidistant starting points at the periphery of specimens and applying an equal number of strokes with a diamond bur to maintain a uniform depth. The grooves were designed with varying lengths to serve as asymmetrical reference markers, facilitating precise alignment between baseline and post-wear measurements.

Specimens were treated with antireflective powder (Occlutec Spray, Rentert, USA), scanned using 3D scanner (DOF—Freedom HD Dental Scanner, DOF Inc, Korea), with accuracy of ≈ 7 μm, then underwent chewing simulation.

### Microhardness test

Surface microhardness was evaluated at baseline and post-simulation (within the resultant wear facets) using a Vickers Hardness Tester (Indentec, Zwick Roell Co., USA). Following ASTM E 384 − 89 standards [33], a load of 200 gf was applied for a dwell time of 10 s. To allow for direct comparison of how the wear process influenced the materials’ surface hardness, indentations were performed in the center of each wear facet.

The indentation diagonals were measured then HVN was calculated by the machine’s software. The average of the hardness measurement was obtained from 5 indentations for each specimen formulation, then the mean value was calculated.

### Surface roughness

Following ISO 25178-2:2012 [34], specimens were evaluated twice (before and after chewing simulation within the wear facets) using non-contact profilometry reconstructed from high-resolution digital micrographs. Images were acquired at 120x magnification (1280 × 1024 pixels) with a stabilized 8-LED circumferential array (CRI ~ 95%) was utilized at a 90° incidence angle (Scope Capture Digital Microscope, China). To ensure quantitative accuracy, the system was spatially calibrated using a certified precision microscopic scale, yielding a lateral resolution of approximately 0.8 μm per pixel. 3D topographic reconstructions were processed via WSxM software (Nanotec Ver 5 develop 4.1, Nanotec, Electronica, SL), utilizing validated algorithms for converting intensity data into height maps. While the system measured areal surface texture (Sa) over a defined field, the results are reported as (Ra) to facilitate direct comparison with established literature. To ensure high-fidelity capture of micro-structural changes, three distinct regions (10µmx 10 μm) were analyzed per specimen, with a vertical resolution threshold of approximately 10 nm, providing sufficient sensitivity to detect matrix recession and filler exposure. The arithmetic means height of the surface was calculated according to the principles of ISO 25,178 for areal surface texture [35]. The three measurements were averaged and used for statistical analysis.

Scanning electron microscopy (SEM) was performed on one representative specimen from each group. The selected specimens were ultrasonically cleaned, air-dried, and sputter-coated with a thin layer of gold to ensure conductivity. SEM examination was carried out at an accelerating voltage of [30 kV] under high vacuum conditions, and micrographs were obtained at different magnifications to assess surface morphology and wear features.

### Three body-wear

Wear resistance was measured according to ISO/TS 14569-2:2001 by chewing simulator. (Chewing simulator CS-4.4, SD Mechatronik GMBH, Germany) for 120,000 cycles at a frequency of 0.96 Hz, which is a widely accepted protocol for simulating approximately six months of clinical service in the oral cavity [32].

**Fig. 2:**
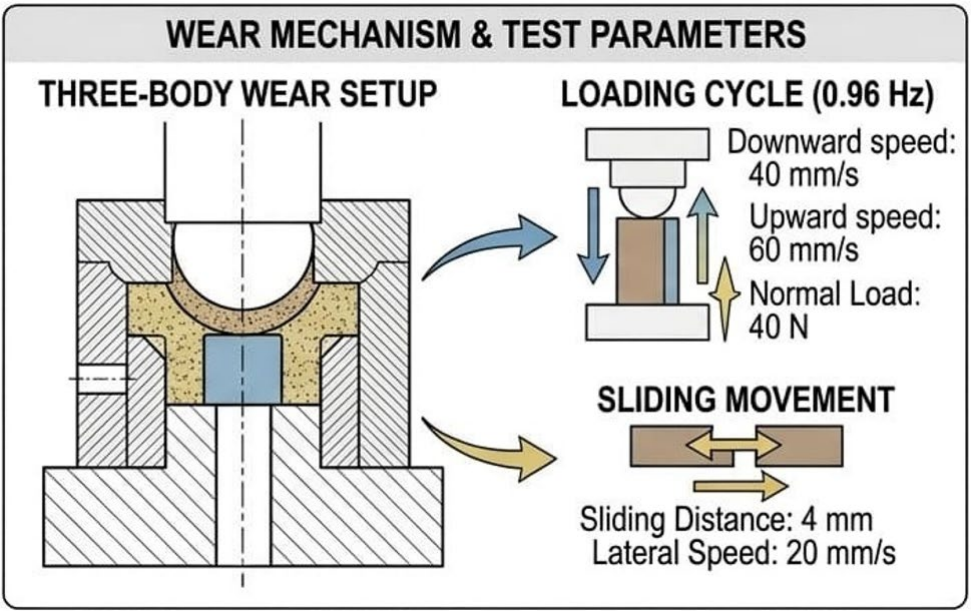
Schematic illustration of 3-body wear setup test

The process of chewing simulation was achieved using pumice/distilled water (20–50µm-30%) as friction medium between steatite ball (4 mm diameter) and specimens, with the following settings (40 N load, 40 mm/s downward speed, sliding distance of 4 mm, 20 mm/s lateral speed and 60 mm/s upward speed). The chewing simulation parameters incorporated both vertical impact and horizontal sliding, which more closely resemble physiological masticatory conditions. Each cycle commenced with a vertical movement of the antagonist toward the specimen (impact phase), followed by a sliding displacement, and finally a vertical lift-off. This dual-action protocol is essential for simulating three-body wear, as the initial impact forces the abrasive slurry into the material surface before the sliding motion. In the chewing simulator, the four test compartments are interconnected, allowing the abrasive slurry to circulate evenly between them. This design ensures that the same amount of abrasive medium is maintained across all specimens during the wear simulation, thereby providing standardized testing conditions for all groups. These conditions were selected to simulate masticatory function, including the representative magnitude and direction of forces acting during posterior functional mastication [36–38]. A pumice-based slurry was selected to establish a standardized three-body wear model. While more abrasive than a typical human food bolus, this ‘accelerated wear’ protocol was utilized to ensure a measurable and statistically significant material response within 120,000 cycles (equivalent to approximately 6 months of clinical service). This approach effectively isolates the intrinsic wear resistance of the polymer matrices and filler-shielding mechanisms, providing a clear performance hierarchy that might be obscured by less aggressive, non-standardized media. While thermal cycling was not performed to isolate the mechanical wear variables. The ball contacted the specimen initially as a point contact, which developed into a circular wear facet as wear cycles progressed (Fig. 2). The wear facet refers to the localized worn surface generated by the contact between the antagonist and the specimen during the chewing simulation. This region was used as the reference area for surface analysis, and was quantitatively assessed by measuring the volumetric material loss.

**Fig. 3:**
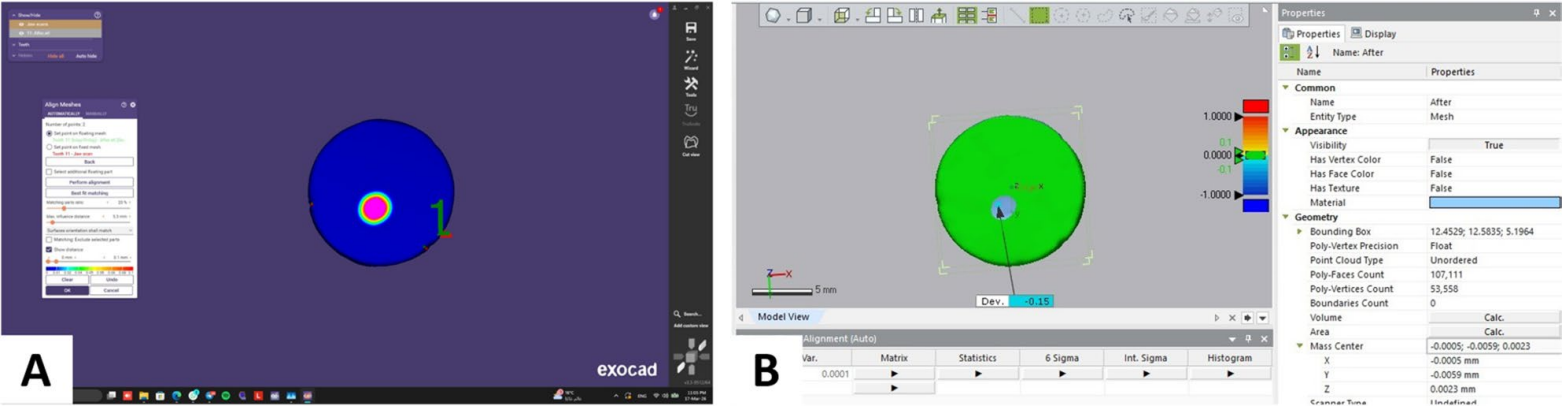
Volumetric wear analysis via digital 3D superimposition. **A** Baseline and post-wear STL files were aligned in Exocad the purple area represents the negative deviation, identifying material loss (the wear facet), **B**) Geomagic inspection view showing the topographical depth map. The color spectrum quantifies material loss

Following chewing simulation, specimens were stored at room temperature for 24 hours to standardize the environmental state, sprayed again and optically rescanned. For analysis, each specimen’s two images were imported to the Exocad program. The exocad program then made it possible to accurately align and superimpose the two 3D surface scans. To eliminate alignment bias, we employed a constrained Best-Fit registration targeting only the stable, unworn peripheral areas of the specimens. This ensured that the ‘unworn’ surfaces remained at zero deviation, accurately isolating the material loss within the wear facet for volumetric calculation. After which Geomagic software (3D Systems, USA) was used for quantitative measurement of wear facets’ volume in cubic millimeters Fig. 3. All scans were performed under standardized conditions, and the same operator conducted the scanning and alignment procedures to reduce variability.

Following the wear simulation, the border of the wear facet was delineated using a fine-tipped permanent marker under 10x magnification. This served as a visual reference to ensure that the subsequent surface roughness scans and microhardness indentations were accurately centralized within the wear track, avoiding the adjacent unworn material.

### Statistical analysis

The data was statistically analyzed by Statistical Package of Social Science (SPSS). Normality of data was confirmed by Shapiro-wilk test (*P* > 0.05). Descriptive statistics were calculated as means and standard deviations. Two- Way Anova was used to evaluate effect of 3 body wear and material type on the tested parameters, followed by pair-wise comparison. For comparing wear volumes between the three groups, One-way Anova followed by post hoc-test was used. For all tests, *P*-value < 0.05 was considered statistically significant. Pearson correlation was performed to quantitatively assess the relationship between wear volume, surface roughness, and hardness values measured before and after the wear simulation. The test was used to determine the strength and direction of the associations between these variables, with significance at the 0.01 level. Following data collection, a retrospective power analysis was conducted using the observed means and standard deviations, yielding a statistical power of > 99%, thereby confirming the adequacy of the sample size to detect significant differences in wear behavior among the tested materials.

## Results

### Wear volume (mm^3^)

One way ANOVA showed significant difference between the groups (F = 1590.9, *P* = 0.000*), with PMMA group showing significantly higher values compared to both composite and PEEK groups (*p* < 0.001*). While comparing composite to PEEK, composite group had significantly lower wear volumes than PEEK (*p* < 0.001*) (Table 2).

**Table 2:** The outcomes of statistical results for wear volumes (mm^3^)

Group	Mean ± SD	F	*P*-Value
PMMA	0.55 ± 0.032 ^**a**^	**1590.9**	< 0.001*
Composite	0.089 ± 0.005 ^**b**^		
PEEK	0.23 ± 0.038 ^**c**^		

* Indicates significance

Different superscript letters indicate significance difference between the groups (Tukey’s HSD, *p* < 0.05)

### Surface roughness

The mean (Sa, reported as Ra; µm) and standard deviations of all groups are presented in (Table 3).

**Table 3:** The outcomes of statistical tests for surface roughness (Sa, reported as Ra; µm)

	Group	Mean ± SD
PMMA^A^	Before wear	0.06 ± 0.004
	After wear	0.34 ± 0.035
Composite^B^	Before wear	0.024 ± 0.003
	After wear	0.18 ± 0.025
PEEK^C^	Before wear	0.042 ± 0.002
	After wear	0.2 ± 0.016

Two-way ANOVA results		
	F	*P*-Value
Material	343.01	< 0.001*
Wear	3823.27	< 0.001*
Material*Wear	158.11	< 0.001*

* Indicates significance

Different uppercase superscript letters ^(A, B, C)^ denote significant pairwise differences between material groups based on Tukey HSD post-hoc testing (*p* < 0.001*)

**Fig. 4:**
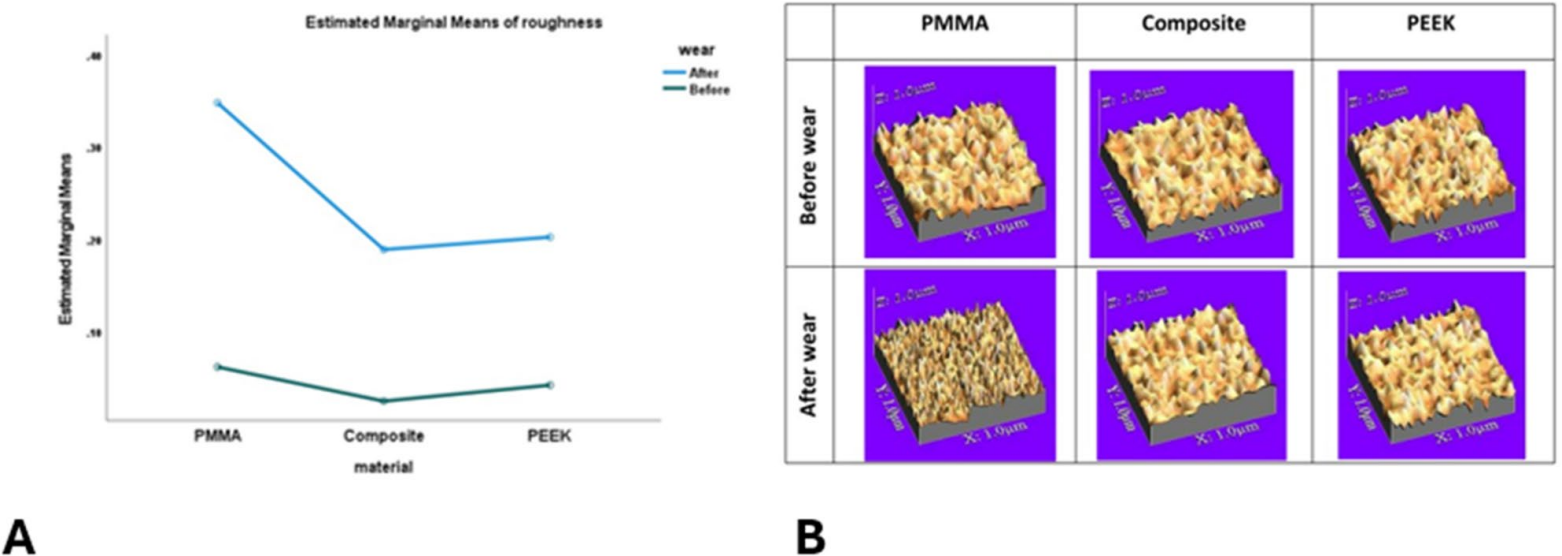
Surface roughness results, **A**: Two-way Anova results, **B**: surface profile of representative samples from each group before and after wear

Two-way Anova results indicated significant interaction between material type and wear on surface roughness values (F = 158.113, *P*-Value < 0.001*) (Fig. 4). Significant main effects were also observed for both the material type (F = 334.016, *p* < 0.001*) and the wear simulation (F = 3823.277, *p* < 0.001*), indicating that both variables fundamentally influenced surface topography. Post-hoc analysis using the Tukey HSD test further revealed that surface roughness differed significantly between all tested material groups (*p* < 0.001*). For the Wear factor, which contained only two levels (Before and After), the significant main effect from the two-way ANOVA was utilized to denote statistical significance, as post-hoc testing is not indicated for factors with fewer than three levels. The roughness values increased by approximately 5.6-fold for PMMA, 7.5-fold for the composite material, and 4.7-fold for PEEK after the wear simulation.

**Fig. 5:**
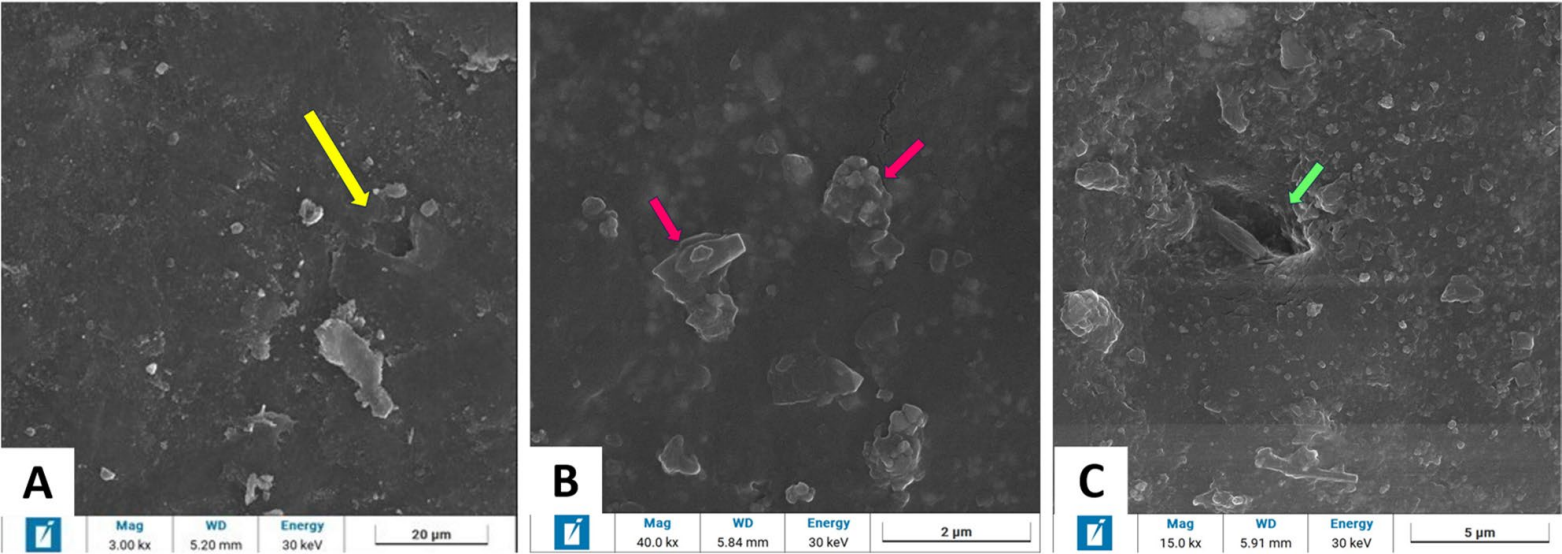
Representative SEM micrographs of the tested materials following wear (**A**) PMMA: showing micro-ploughing (yellow arrow), (**B**) Nano-ceramic Composite: revealing the inorganic ceramic particles (red arrows) remain largely intact, (**C**) PEEK: showing localized plastic deformation and shallow pits (green arrows)

The SEM images (Fig. 5) show that for PMMA, the surface exhibits irregular craters and significant loss of material continuity (micro-ploughing). For composite specimen, the filler particles act as “hard stops” against the abrasives. The resin matrix between them might recede slightly, but the fillers prevent deep scratching. The PEEK’s surface shows localized plastic deformation It displays a more “leathery” wear pattern, characteristic of high-toughness polymers.

### Microhardness

**Fig. 6:**
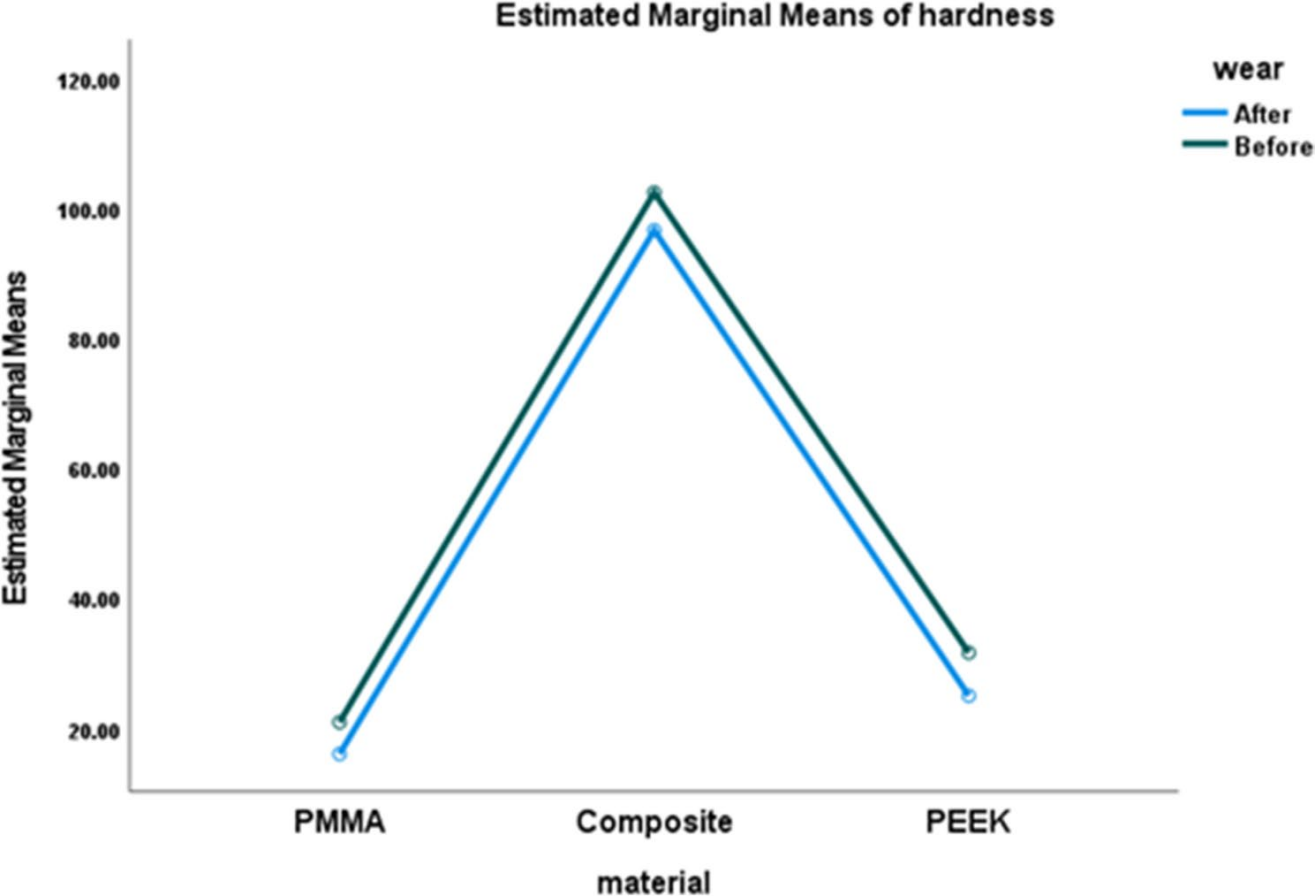
Comparison of the three groups regarding microhardness (VHN)

The descriptive VHN data of all groups are presented in (Table 4). Two-way Anova results indicated non-significant interaction between material type and wear on microhardness values (F = 0.111, *P*-Value = 0.89) (Fig. 6). A highly significant main effect was found for the material type (F = 1158.28, *P*-Value < 0.001*). The wear simulation also showed a significant main effect (F = 14.77, *P*-Value < 0.001*). (Table 4) for the material factor, Post-hoc comparisons using the Tukey HSD test revealed that the surface hardness differed significantly between all tested material groups (*p* < 0.001). For the Wear factor, which contained only two levels (Before and After), the significant main effect from the two-way ANOVA was utilized to denote statistical significance, as post-hoc testing is not indicated for factors with fewer than three levels. The percentage reduction in hardness was approximately 23.5% for PMMA, 9.5% for the composite material, and 21% for PEEK.

**Table 4:** The outcomes of statistical tests for microhardness

	Group	Mean ± SD
PMMA^A^	Before wear	20.79 ± 0.76
	After wear	15.91 ± 0.65
Composite^B^	Before wear	106.63 ± 2.92
	After wear	96.44 ± 0.66
PEEK^C^	Before wear	31.47 ± 0.93
	After wear	24.85 ± 0.49

Two-way ANOVA results		
	F	*P*-Value
Material	1158.28	< 0.001*
Wear	14.77	< 0.001*
Material*Wear	0.111	0.895

* Indicates significance

Different uppercase superscript letters ^(A, B, C)^ denote significant pairwise differences between material groups based on Tukey HSD post-hoc testing (*p* < 0.001*)

### Correlation results

Pearson correlation analysis revealed a significant negative correlation between baseline hardness and wear volume (*r* = -0.760, *p* < 0.001*). The calculated coefficient of determination (*R*^2^ = 0.58) indicates that surface hardness is a moderately strong predictor of abrasive resistance, accounting for 58% of the volumetric variance across the tested CAD/CAM materials. Surface roughness demonstrated an even more robust positive correlation (*r* = 0.948, *R*^2^ = 0.90, *p* < 0.001*). This indicates that surface topography changes account for 90% of the variance in material loss, suggesting that the progression of surface degradation is a highly accurate predictor of total wear volume in these CAD/CAM resins. The correlation results are presented in Table 5.

**Table 5:** Pearson correlation results between tested properties

Property	Wear volume	
	Pearson correlation coefficient	Significance
Surface hardness (Baseline)	-0.760	0.000*
Surface hardness (After wear)	-0.791	0.000*
Surface roughness (Baseline)	0.948	0.000*
Surface roughness (After wear)	0.907	0.000*

*. Correlation is significant at the 0.01 level

## Discussion

The primary objective of this study was to evaluate the wear volume of CAD/CAM PMMA, nano-ceramic composites, and PEEK under 3-body wear simulation. By targeting measurements specifically within the wear facets, this study provided an assessment of wear effect on surface roughness and hardness. In vitro investigations offer greater control over wear mechanisms compared to clinical research, which have constraints such as complex technique and challenging measurement and analysis [39].

Since wear is a complex process, it is more challenging to measure it directly in the setting of the mouth. Wear simulation tools have been developed to examine the wear behavior of dental restorative materials [40, 41]. Clinically, three-body wear may be more significant than two-body wear because restorative materials have less direct contact with opposing teeth or restorations compared to food boluses or toothpaste. In a previous study by McCabe et al., they concluded that wear of resin matrix substrates is related to relative dentine abrasivity (RDA) of the abrasive particles [42]. Hereby in this study, pumice was used as the abrasive material to simulate the worst-case scenario for resin composites as it has high RDA [39, 43].

Steatite balls were used as the antagonist material since it has been revealed that it has abrasive wear characteristics comparable to enamel and composite materials [44]. By using a standardized ceramic antagonist instead of natural teeth, a uniform contact geometry was maintained for all specimens. This approach reduced variability arising from the anisotropic structure and natural differences of human enamel, allowing the intrinsic wear resistance of the CAD/CAM polymer materials to be evaluated more reliably as the main variable. For one year of chewing in the oral cavity, a minimum of 240,000 to 250,000 cycles is needed, resulting in 120,000 to 130,000 cycles offer a six-month period [45]. The model is designed to simulate generalized occlusal wear. By applying a vertical load together with a sliding motion in the presence of an abrasive medium, it replicates the grinding phase of mastication.

Volumetric wear loss was computed in this investigation by superimposing the specimens before and after testing to produce three-dimensional pictures. These images were then imported into the three-dimensional analysis program. To ensure high-precision alignment and mitigate potential hardware scanning noise, a localized ‘best-fit’ registration algorithm was employed, utilizing only the non-functional, stable reference surfaces of each specimen. This standardized subtraction methodology is considered the gold standard for isolating material loss in dental tribology simulations [46]. 3-D photographs with an estimated difference of 20 mm between pre- and post-testing were considered acceptable [47, 48].

An ideal restorative material should match the functional wear characteristics of natural enamel—exhibiting sufficient resistance to material loss while producing minimal abrasion of opposing dentition [49]. In the present study, interpretation of the wear data required rejection of the first null hypothesis, as the three evaluated materials exhibited significantly different wear volumes. Wear behavior is determined by a combination of material properties—such as hardness, fracture toughness, porosity, and surface quality—as well as external factors including the presence of staining or abrasive media and the frictional resistance of the antagonist [50]. Despite both the nanohybrid composite and the ceramic-reinforced PEEK (BioHPP) incorporating nanoceramic fillers, they exhibited distinct wear patterns. This variation is likely due to fundamental differences in their filler-to-matrix ratios and polymer structures. The nano-ceramic composite features a high-density filler system (≈ 86%), creating a shield of closely packed inorganic particles that protects the resin matrix from abrasive degradation [51]. In contrast, BioHPP contains a lower filler concentration (≈ 20%), where the ceramic particles primarily serve to increase the elastic modulus of the PEEK matrix. The moderate wear resistance of BioHPP is likely a synergistic result of this ceramic reinforcement and the inherent toughness of its semi-crystalline thermoplastic matrix [50]. This structural complexity also explains why PEEK outperformed PMMA. As an unfilled, linear polymer with a polar, hydrophilic nature, PMMA is more susceptible to water sorption and hydrolytic degradation, making its surface significantly more vulnerable to abrasive loss during wear cycles. In addition, the combination of lower surface hardness and the lack of reinforcing fillers in the PMMA group together with the high surface roughness that may cause increased friction and wear. It can also cause superficial cracks to form and spread, which could weaken a restoration [52].

The findings should be interpreted as a comparative durability hierarchy rather than absolute predictors of intraoral wear. While the oral environment is complex and includes factors such as salivary lubrication, pH variation, enzymatic activity, and thermal cycling, the mechanical “stress test” provided by the steatite–slurry model enables controlled comparison of material behavior under standardized abrasive conditions. The strong correlation observed between surface roughness and volumetric loss (*R*² = 0.90) suggests that topographical degradation is closely associated with material loss within this model and may serve as a useful indicator of relative wear performance. Accordingly, the results indicate that highly filled nano-ceramic composites demonstrate greater resistance to wear compared to unfilled PMMA under the tested conditions. However, clinical performance may vary depending on patient-specific factors and oral environment variability.

These findings were confirmed by the correlation results, which are consistent with the established tribological principles and Archard’s wear law, where harder materials generally show improved resistance to abrasive wear, while increased surface roughness can facilitate abrasive interactions and accelerate material removal [53].

The greatest volumetric loss related to PMMA, is consistent with the data reported by a finding reported by Vohra et al., who assessed two-body wear in several CAD/CAM restorative materials [29].

Additional issues with CAD-CAM resin blocks include surface roughness and biofilm growth, which can affect the material’s hygienic and aesthetic qualities. Researchers have observed that studies suggest resin composites may encourage bacterial growth more than other restorative materials as ceramics, resulting in greater biofilm buildup [54, 55].

PEEK showed less roughness than PMMA, but greater roughness than the indirect composite. The fine filler particle size promotes a uniform material microstructure, which likely contributed to the relatively low surface roughness (Ra) observed after wear, even though the material exhibited higher volumetric loss than the composite. PMMA demonstrated the highest roughness among all groups both before and after wear. This causes the second null hypothesis to be rejected. To ensure comparability, a standardized polishing protocol was employed so that all specimens achieved a baseline roughness (Ra) well below the 0.2 μm critical threshold for bacterial retention reported by Bollen et al. [56] Initial values ranged from 0.024 ± 0.003 μm for composite to 0.06 ± 0.004 μm for PMMA; while statistically distinct, these values represent a clinically equivalent smooth state. The subsequent wear simulation triggered a topographical degradation of up to 5.6-fold in the PMMA group, suggesting that final outcomes were dictated by the materials’ bulk tribological resistance rather than minor initial variations.

Furthermore, the significant positive correlation between baseline roughness and volumetric wear indicates that a material’s ‘polishability’ serves as a predictive sentinel for its structural integrity. This relationship is fundamentally governed by the filler-shielding mechanism: the high inorganic loading (86%) of the nanohybrid composite facilitates a more refined initial surface and provides a rigid scaffold that protects the resin matrix from the micro-ploughing and abrasive fatigue typical of long-term material loss. Conversely, the unfilled PMMA matrix lacks this protective architecture, leading to both a higher baseline Ra and accelerated volumetric degradation. Ultimately, these results confirm that wear performance is a direct reflection of the materials’ intrinsic reinforcement strategies rather than a legacy of the initial surface preparation.

According to Jones et al., a patient’s tongue can identify the Ra of 0.25–0.5 μm [57]. In a study by Koizumi et al., they compared surface roughness behaviors of different composite blocks with different filler contents before and after tooth brushing simulation and found that the polymeric matrix, filler size, filler shape, and silanization all affect surface roughness and consequently gloss reduction [58].

PEEK demonstrates mechanical behavior that approximates that of natural hard tissues, particularly in terms of elastic modulus and stress distribution, which contributes to its favorable tribological performance. This biomimetic mechanical compatibility enables PEEK-based materials—especially reinforced formulations—to exhibit wear characteristics comparable to enamel, including reduced abrasiveness and the ability to dissipate occlusal forces through elastic deformation and self-lubricating mechanisms. Consequently, PEEK is widely regarded as a promising candidate for applications requiring balanced wear resistance and preservation of opposing dentition [59, 60].

Surface hardness testing is helpful for assessing composites’ wear resistance properties [61, 62]. In this investigation, composite outperformed PEEK and PMMA exhibiting the highest hardness values before and after chewing simulation. The wear effect on hardness could be owing to the high abrasivity of the pumice particles that may led to surface fatigue or microcracks, thus decreasing the surface hardness [63]. Therefore; the third null hypothesis was rejected.

The presence of water in combination with pumice promotes water uptake by the polymeric components, which can induce hydrolysis of the silane coupling agents and impair the interfacial adhesion between the matrix and the filler. Consequently, the material’s surface hardness may decrease as filler particles become dislodged from the surface [64].

The findings of this study provide empirical validation for the clinical application of CAD/CAM materials based on their mechanical response to wear as follows; Nano-ceramic composites demonstrated superior wear resistance and surface stability, supporting their use in permanent load-bearing areas to prevent loss of vertical dimension, PEEK’s stable surface roughness confirms its suitability for RPD frameworks and abutments where soft-tissue compatibility is a priority. Finally, the high wear rate of PMMA reinforces its designation as a short-term restorative material, especially in patients with parafunctional habits.

Clinicians are strongly recommended to strictly adhere to the manufacturer’s indicated use for each material. Selection should be based on the intended clinical duration and the anticipated occlusal stress of the specific site, as the degree of surface degradation and volumetric loss is highly material-dependent.

A limitation of the present study is that the evaluation focused primarily on the surface characteristics of the tested CAD/CAM materials after three-body wear simulation. Although surface roughness and surface alterations are important indicators of wear behavior and may influence plaque accumulation, aesthetics, and material longevity, other clinically relevant outcomes were not assessed. In addition, baseline roughness standardization was not fully controlled experimentally, which may introduced variability. This study focused exclusively on the degradation of the restorative materials without assessing post-wear functional outcomes, such as residual flexural strength. The wear and post-test surface condition of the antagonists were not measured, which necessitates caution when extrapolating these findings to complex, multi-axial clinical loading scenarios. However, the use of standardized steatite spheres limited potential geometric changes. In addition, their hardness (680 HV) and wear characteristics provide a standardized, reproducible proxy for human enamel (330 HV) ensuring the abrasive challenge was clinically representative [65].

The present investigation assessed the material behavior after a single fatigue interval (6 months). Evaluating multiple fatigue cycle intervals could provide additional insight into the progressive changes in surface characteristics over time. Another limitation of this study is the high abrasivity of the pumice slurry compared to a typical human bolus. This setup represents an accelerated wear model designed to highlight structural differences in material resistance over a compressed period. In a clinical setting, where the diet may be less abrasive, the volumetric loss for materials might be less severe. While retrospective power analysis confirmed the adequacy of the sample size, we acknowledge that these in vitro findings remain exploratory in relation to clinical performance, where biological variables such as saliva chemistry and variable chewing patterns may influence materials. Although the in-vitro model offers controlled and reproducible testing conditions, it does not replicate the biomechanical environment of the oral cavity, particularly the cushioning effect of the periodontal ligament and the elastic support provided by the alveolar bone under functional loads [47, 48]. Nonetheless, despite these limitations, in-vitro wear simulations remain essential for generating early predictive data on material performance and for guiding the clinical evaluation of newly introduced restorative products within a practical timeframe. Future investigations should incorporate parameters such as residual mechanical strength after wear, crack initiation or propagation within the material structure, and the potential wear effect on antagonist surfaces utilizing SEM and profilometry to provide a complete mapping of the wear interface to provide additional insight into the functional performance of these materials. Moreover, investigation of longer chewing cycles would better simulate the cumulative effects of long-term oral function.

## Conclusion

Within the limitations of this study, it can be concluded that three-body wear simulation significantly degrades the surface roughness and microhardness of CAD/CAM materials, with the degree of degradation being highly material-dependent and significantly correlates to its baseline microhardness. Furthermore, material microstructure played a key role in determining the surface characteristics within the wear facet. The findings validate the manufacturers’ indications for these materials. Clinicians should strictly adhere to these guidelines, utilizing nano-composites for definitive restorations and reserving PMMA for short-term provisional use.

## Data Availability

On reasonable request, the datasets utilized and/or analyzed during the present study are accessible from the corresponding author.

## Abbreviations

Ra: Average roughness
CAD/CAM: Computer aided design/computer aided manufacturing
PMMA: Polymethyl methacrylate
PEEK: Polyether ether ketone
RDA: Relative dentine abrasivity
RPD: Removable partial denture

## References

[1] D’Arcangelo C, Vanini L, Rondoni GD, Pirani M, Vadini M, Gattone M, De Angelis F. Wear properties of a novel resin composite compared to human enamel and other restorative materials. Oper Dent. 2014;39:612–8. 25084103 10.2341/13-108-L

[2] Stober T, Lutz T, Gilde H, Rammelsberg P. Wear of resin denture teeth by two-body contact. Dent Mater. 2006;22:243–9. 16084585 10.1016/j.dental.2005.03.009

[3] Ghazal M, Yang B, Ludwig K, Kern M. Two-body wear of resin and ceramic denture teeth in comparison to human enamel. Dent Mater. 2008;24:502–7. 17688934 10.1016/j.dental.2007.04.012

[4] Gwon B, Bae EB, Lee JJ, Cho WT, Bae HY, Choi JW, Huh JB. Wear characteristics of dental ceramic CAD/CAM materials opposing various dental composite resins. Mater (Basel). 2019;12:1839. 10.3390/ma12111839 PMC6600963 31174298

[5] Tsujimoto A, Barkmeier WW, Fischer NG, Nojiri K, Nagura Y, Takamizawa T, Latta MA, Miazaki M. Wear of resin composites: current insights into underlying mechanisms, evaluation methods and influential factors. Jpn Dent Sci Rev. 2018;54:76–87. 29755618 10.1016/j.jdsr.2017.11.002 PMC5944074

[6] Heintze SD, Ilie N, Hickel R, Reis A, Loguercio A, Rousson V. Laboratory mechanical parameters of composite resins and their relation to fractures and wear in clinical trials-A systematic review. Dent Mater. 2017;33:e101–14. 27993372 10.1016/j.dental.2016.11.013

[7] Grau A, Stawarczyk B, Roos M, Theelke B, Hampe R. Reliability of wear measurements of CAD-CAM restorative materials after artificial aging in a mastication simulator. J Mech Behav Biomed Mater. 2018;86(4):185–90. 29986292 10.1016/j.jmbbm.2018.06.030

[8] Lambrechts P, Debels E, Van Landuyt K, Peumans M, Van Meerbeek B. How to simulate wear? Overview of existing methods. Dent Mater. 2006;22(8):693–701. 16712913 10.1016/j.dental.2006.02.004

[9] Dionysopoulos D, Gerasimidou O. Wear of contemporary dental composite resin restorations: a literature review. Restor Dent Endod. 2021;46(2):1–13. 10.5395/rde.2021.46.e18 PMC8170387 34123754

[10] Tsujimoto A, Barkmeier W, Takamizawa T, Latta M, Miyazaki M. Influence of thermal stress on simulated localized and generalized wear of nanofilled resin composites. Operat Dent. 2018;43(4):380–90. 10.2341/16-206-L 29949478

[11] Tsujimoto A, Barkmeier W, Takamizawa T, Latta M, Miyazaki M. Influence of thermal cycling on flexural properties and simulated wear of computer aided design/computer-aided manufacturing resin composites. Operat Dent. 2017;42(1):101–10. 10.2341/16-046-L 27802120

[12] Lauvahutanon S, Takahashi H, Oki M, Arksornnukit M, Kanehira M, Finger W, CAM. Dent Mater J. 2015;34(4):495–502. 26235715 10.4012/dmj.2014-293

[13] Poggio C, Lombardini M, Gaviati S, Chiesa M. Evaluation of Vickers hardness and depth of cure of six composite resins photo-activated with different polymerization modes. J Conserv Dent. 2012;15(3):237–24. 22876009 10.4103/0972-0707.97946 PMC3410332

[14] Zafar M, Ahmed N. Effects of wear on hardness and stiffness of restorative dental materials. Life Sci J. 2014;11(10s):11–8.

[15] DeLong R, Pintado MR, Douglas WH, Fok AS, Wilder AD, Swift EJ, Bayne SC. Wear of a dental composite in an artificial oral environment: A clinical correlation. J Biomed Mater Res B Appl Biomater. 2012;100B(8):2297–306. 10.1002/jbm.b.32801 22997090

[16] Say E, Civelek A, Nobecourt A, Ersoy M, Guleryuz C. Wear and microhardness of different resin composite materials. Oper Dent. 2003;28(5):628–34. 14531611

[17] Kakaboura A, Fragouli M, Rahiotis C, Silikas N. Evaluation of surface characteristics of dental composites using profilometry, scanning electron, atomic force microscopy and gloss-meter. J Mater Sci Mater Med. 2007;18:155–63. 17200827 10.1007/s10856-006-0675-8

[18] Hosoya Y, Shiraishi T, Odatsu T, Ogata T, Miyazaki M, Powers JM. Effects of specular component and polishing on color of resin composites. J Oral Sci. 2010;52:599–607. 21206163 10.2334/josnusd.52.599

[19] Ergücü Z, Türkün LS. Surface roughness of novel resin composites polished with one-step systems. Oper Dent. 2007;32:185–92. 17427829 10.2341/06-56

[20] Cavalcante LM, Masouras K, Watts DC, Pimenta LA, Silikas N. Effect of nanofillers size on surface properties after toothbrush abrasion. Am J Dent. 2009;22:60–4. 19281115

[21] Musanje L, Ferracane JL, Ferracane LL. Effects of resin formulation and nanofiller surface treatment on in vivo wear of experimental hybrid resin composite. J Biomed Mater Res B Appl Biomater. 2006;77:120–25. 16184536 10.1002/jbm.b.30400

[22] Al-Dwairi ZN, Tahboub KY, Baba NZ, Goodacre CJ. A Comparison of the Flexural and Impact Strengths and Flexural Modulus of CAD/CAM and Conventional Heat-Cured Polymethyl Methacrylate (PMMA). J Prosthodont. 2020;29(4):341–49. 29896904 10.1111/jopr.12926

[23] Limírio J, Gomes J, Rezende M, Lemos C, Rosa C, Pellizzer E. Mechanical properties of polymethyl methacrylate as a denture base: Conventional versus CAD-CAM resin - A systematic review and meta-analysis of in vitro studies. J Prosthet Dent. 2022;128(6):1221–29. 34030891 10.1016/j.prosdent.2021.03.018

[24] Edelhoff D, Beuer F, Schweiger J, Brix O, Stimmelmayr M, Güth JF. CAD/CAM generated high-density polymer restorations for the pretreatment of complex cases: a case report. Quintessence Int. 2012;43:457–67. 22532953

[25] Rexhepi I, Santilli M, D’Addazio G, Tafuri G, Manciocchi E, Caputi S, Sinjari B. Clinical Applications and Mechanical Properties of CAD-CAM Materials in Restorative and Prosthetic Dentistry: A Systematic Review. J Funct Biomater. 2023;14(8):431. 37623675 10.3390/jfb14080431 PMC10455074

[26] Garza L, Crooke E, Vallés M, Soliva J, Rodríguez X, Rodeja M, Miguel Roig. Evaluation of Polymethyl Methacrylate as a Provisional Material in a Fully Digital Workflow for Immediate-Load Complete-Arch Implant-Supported Prostheses over Three Months. Materials. 2025;18(3):562. 39942227 10.3390/ma18030562 PMC11818157

[27] Angelara K, Bratos M, Sorensen J. Comparison of strength of milled and conventionally processed PMMA complete-arch implant-supported immediate interim fixed dental prostheses. J Prosthet Dent. 2023;129:221–27. 34158174 10.1016/j.prosdent.2021.04.025

[28] Stawarczyk B, Sener B, Trottmann A, Roos M, Özcan M, Hämmerle CH. Discoloration of manually fabricated resins and industrially fabricated CAD/CAM blocks versus glass-ceramic: effect of storage media, duration, and subsequent polishing. Dent Mater J. 2012;31:377–83. 22673470 10.4012/dmj.2011-238

[29] Vohra M, Wadhwani V, Khushali K. Comparative Evaluation of Two Body Wear of PEEK vs Milled PMMA vs Indirect Composite After Chewing Simulation- An In Vitro Study. Nanatechnol Perceptions. 2024;20(9):1094–101.

[30] Maier E, Grottschreiber C, Knepper I, Opdam N, Petschelt A, Loomans B, Lohbauer U. Evaluation of wear behavior of dental restorative materials against zirconia in vitro. Dent Mater. 2022;38(5):778–88. 35459553 10.1016/j.dental.2022.04.016

[31] Heintze SD, Reichl FX, Hickel R. Wear of dental materials: clinical significance and laboratory wear simulation methods – a review. Dent Mater J. 2019;38(3):343–53. 30918233 10.4012/dmj.2018-140

[32] International Organization for Standardization. Dental materials — Guidance on testing of wear. Part 2: Wear by two- and/or three body contact. 2001. ISO/TS 14569-2:2001.

[33] Standard Test Method for Microindentation Hardness of Materials. Denture-Based Polymer. ASTM International; 2017.

[34] International Organization for Standardization. Geometrical Product Specification (GPS) - Surface Texture: Area - Part 2: Terms, Definitions, Surface Texture Parameters and Techniques. 2012; ISO25178:2012(2).

[35] Aver’Yanova I, Bogomolov D, Poroshin V. ISO 25178 standard for three-dimensional parametric assessment of surface texture. Russ Eng Res. 2017;37(6):513–6.

[36] Kielbassa AM, Oehme EP, Shakavets N, Wolgin M. In vitro wear of (resin-coated) high-viscosity glass ionomer cements and glass hybrid restorative systems. J Dent. 2021;105:103554. 33309807 10.1016/j.jdent.2020.103554

[37] Koottathape N, Takahashi H, Iwasaki N, Kanehira M, Finger WJ. Two-and three-body wear of composite resins. Dent Mater. 2012;28(12):1261–70. 23083806 10.1016/j.dental.2012.09.008

[38] Koletsi D, Iliadi A, Eliades T, Eliades G. Vitro Simulation and In Vivo Assessment of Tooth Wear: A Meta-Analysis of In Vitro and Clinical Research. Mater (Basel). 2019;12(21):3575. 10.3390/ma12213575 PMC6862526 31683544

[39] Sripetchdanond J, Leevailoj C. Wear of human enamel opposing monolithic zirconia, glass ceramic, and composite resin: an in vitro study. J Prosthet Dent. 2014;112(5):1141–50. 24980740 10.1016/j.prosdent.2014.05.006

[40] Shimane T, Endo K, Zheng JH, Yanagi T, Ohno H. Wear of opposing teeth by posterior composite resins–evaluation of newly developed wear test methods. Dent Mater J. 2010;29(6):713–20. 21099153 10.4012/dmj.2008-031

[41] McCabe J, Molyvda S, Rolland S, Rusby S, Carrick T. Two-and three-body wear of dental restorative materials. Int Dent J. 2002;52:406–16.

[42] Bekhiet A, Katamish H, Salah T. The effect of two body wear on CAD/CAM PEEK against Monolithic Zirconia, Lithium Disilicate, and Tooth Enamel; An in vitro study. Egypt Dent J. 2022;68(3):2691–700.

[43] Chong BJ, Thangavel AK, Rolton SB, Guazzato M, Klineberg IJ. Clinical and laboratory surface finishing procedures for zirconia on opposing human enamel wear: A laboratory study. J Mech Behav Biomed Mater. 2015;50:93–103. 26116957 10.1016/j.jmbbm.2015.06.007

[44] Shortall AC, Hu XQ, Marquis PM. Potential countersample materials for in vitro simulation wear testing. Dent Mat. 2002;18(3):246–54. 10.1016/s0109-5641(01)00043-4 11823017

[45] Rayyan MM, Aboushelib M, Sayed NM, Ibrahim A, Jimbo R. Comparison of interim restorations fabricated by CAD/CAM with those fabricated manually. J Prosthet Dent. 2015;114(3):414–9. 26001490 10.1016/j.prosdent.2015.03.007

[46] O’Toole S, Osnes C, Bartlett D, Keeling A. Investigation into the validity of WearCompare, a purpose-built software to quantify erosive tooth wear progression. Dent Mater. 2019;35(10):1408–14. 31402133 10.1016/j.dental.2019.07.023

[47] Flores-Ferreyra BI, Argueta-Figueroa L, Torres-Rosas R, Carrasco-Gutiérrez RG, Casillas-Santana MA. de Los Angeles Moyaho-Bernal M. Dental human enamel wear caused by ceramic antagonists: A systematic review and network meta-analysis. J Prosthodont Res. 2025;69(2):153–62. 38925985 10.2186/jpr.JPR_D_23_00263

[48] Mao Z, Beuer F, Hey J, Schmidt F, Sorensen JA, Prause E. Antagonist enamel tooth wear produced by different dental ceramic systems: A systematic review and network meta-analysis of controlled clinical trials. J Dent. 2024;142:104832. 38211687 10.1016/j.jdent.2024.104832

[49] Soriano-Valero S, Román-Rodriguez J-L, Agustín-Panadero R, Bellot-Arcís C, Fons-Font A, Fernández-Estevan L. Systematic review of chewing simulators: Reality and reproducibility of in vitro studies. J Clin Exp Dent. 2020;12(12):e1189. 33282141 10.4317/jced.57279 PMC7700780

[50] Li K, Meng M, Li C, Liu Y, Liu H, Bai S, Zhong S, Li M, Chen L, Tian M, Niu L, Fang M. Comparative study of wear properties of CAD/CAM PEEK materials, resin ceramics and dental enamel under simulated chewing conditions. J Mech Behav Biomed Mater. 2025;168:107012. 40279742 10.1016/j.jmbbm.2025.107012

[51] Iyer RS, Suchitra SR, Hegde D, Coutinho CA, Priya A. BioHPP: Properties and Applications in Prosthodontics a review. J Res Dentistry. 2019;7(4):72–6 .

[52] Singh B, Jain S, Bhasin N, Kaur J, Borse P, Longkumer P. Comparative Evaluation of Surface Roughness, Wettability, and Hardness of Conventional, Heat-Polymerized, Computer-Aided Designed and Milled, and Three-Dimensionally Printed Polymethyl Methacrylate Denture Base Resins: An In Vitro Study. Cureus. 2025;17(5):e85008. 40585711 10.7759/cureus.85008 PMC12205262

[53] Dejak BD, Langot C, Krasowski M, Klich M. Evaluation of Hardness and Wear of Conventional and Transparent Zirconia Ceramics, Feldspathic Ceramic, Glaze, and Enamel. Materials. 2024;17(14):3518. 39063809 10.3390/ma17143518 PMC11278436

[54] Almuhayya S, Alshahrani R, Alsania R, Albassam A, Alnemari H, Babaier R. Biofilm Formation on Three High-Performance Polymeric CAD/CAM Composites: An In Vitro Study. Polymers. 2025;17:676. 40076168 10.3390/polym17050676 PMC11902371

[55] Vulovi´c S, Nikoli´c-Jakoba N, Radunovi´c M, Petrovi´c S, Popovac A, Todorovi´c M. Mili´c-Lemi´c A. Biofilm Formation on the Surfaces of CAD/CAM Dental Polymers. Polymers. 2023;15:2140. 37177285 10.3390/polym15092140 PMC10181064

[56] Bollen CM, Papaioanno W, Van Eldere J, Schepers E, Quirynen M, van Steenberghe D. The influence of abutment surface roughness on plaque accumulation and peri-implant mucositis. Clin Oral Implants Res. 1996;7(3):201–11. 9151584 10.1034/j.1600-0501.1996.070302.x

[57] Jones CS, Billington RW, Pearson GJ. The in vivo perception of roughness of restorations. Br Dent J. 2004;196(1):42–5. 14966503 10.1038/sj.bdj.4810881

[58] Koizumi H, Saiki O, Nogawa H, Hiraba H, Okazaki T, Matsumura H. Surface roughness and gloss of current CAD/CAM resin composites before and after toothbrush abrasion. Dent Mater J. 2015;34(6):881–7. 26632238 10.4012/dmj.2015-177

[59] Li K, Meng M, Li C, Liu Y, Liu H, Bai S, Zhong S, Li M, Chen L, Tian M, Niu L. Ming Fang. Comparative study of wear properties of CAD/CAM PEEK materials, resin ceramics and dental enamel under simulated chewing conditions. J Mech Behav Biomed Mater. 2025;168:1751–6161. 10.1016/j.jmbbm.2025.107012 40279742

[60] Kandula UKR, Monika D, Verma PC, Rathi A, Saravanan P. A Comprehensive Review on Manufacturing and Characterization of Polyetheretherketone Polymers for Dental Implant Applications. 3D Print Addit Manuf. 2024;11(4):1441–61. 10.1089/3dp.2023.0064 PMC11443112 39360128

[61] Murakami M. Surface properties of an indirect composite polymerized with five laboratory light polymerization systems. J Oral Sci. 2009;51(2):215–21. 19550089 10.2334/josnusd.51.215

[62] Mandikos MN, McGivney GP, Davis E, Bush PJ, Carter JM. A comparison of the wear resistance and hardness of indirect composite resins. J Prosthet Dent. 2001;85(4):386–95. 11319537 10.1067/mpr.2001.114267

[63] Hussain HF, Hill RG, Gillam DG. Quantification of Tooth Wear by Selected Desensitizing Polishing Pastes Using White Light Profilometry. J Dent Maxillofacial Res. 2020;3(4):1–4.

[64] Bayraktar ET, Türkmen C, Atali PY, Tarçin B, Korkut B, Yaşa B. In-vitro evaluation of wear characteristics, microhardness and color stability of dental restorative CAD/CAM materials. Dent Mater J. 2024;43(1):74–83. 38072413 10.4012/dmj.2023-071

[65] Preis V, Weiser F, Handel G, Rosentritt M. Wear performance of monolithic dental ceramics with different surface treatments. Quintessence Int. 2013;44(5):393–405. 10.3290/j.qi.a29151 23479579

